# Supplementary material for: L-cysteine transporter-PCR to detect hydrogen sulfide-producing Campylobacter fetus
Source: PeerJ. 2019 Nov 5;7:e7820. doi: 10.7717/peerj.7820 (PMC6839519; doi:10.7717/peerj.7820)
Supplement: Supplemental Information 3 — Contingency table and calculation of the Cohen’s Kappa Coefficient. Data set from Table 2 comprising 41 strains with reported-H2S production test was included in the analysis. The analysis showed a perfect agreement (ĸ = 1) (https://idostatistics.com/cohen-kappa-free-calculator/). [file peerj-07-7820-s003.docx]

|  | **H_2_S production deduced from *in silico* L-Cys-PCR** | |
| --- | --- | --- |
| **H_2_S production test** | + | - |
| + | 36 | 0 |
| - | 0 | 5 |
